# Rab8a/SNARE complex activation promotes vesicle anchoring and transport in spinal astrocytes to drive neuropathic pain

**DOI:** 10.17305/bb.2024.10441

**Published:** 2024-10-01

**Authors:** Yunqiao Xiao, Gengyi Wang, Guiqiong He, Wanxiang Qin, Ying Shi

**Affiliations:** 1 University-Town Hospital of Chongqing Medical University, Chongqing, China; 2 Institute of Neuroscience, Chongqing Medical University, Chongqing, China; 3 Department of Pain Care, Southwest Hospital, Army Medical University, Chongqing, China; 4 Department of Pain Care, The First Affiliated Hospital of Chongqing Medical University, Chongqing, China

**Keywords:** Neuropathic pain (NPP), astrocytes, Rab8a, vesicular transport, SNARE proteins

## Abstract

Neuropathic pain (NPP) remains a clinically challenging condition, driven by the activation of spinal astrocytes and the complex release of inflammatory mediators. This study aimed to examine the roles of Rab8a and SNARE complex proteins in activated astrocytes to uncover the underlying mechanisms of NPP. The research was conducted using a rat model with chronic constriction injury (CCI) of the sciatic nerve and primary astrocytes treated with lipopolysaccharide (LPS). Enhanced expression of Rab8a was noted specifically in spinal dorsal horn astrocytes through immunofluorescence (IF). Electron microscopy (EM) observations showed increased vesicular transport and exocytic activity in activated astrocytes, which was corroborated by elevated levels of inflammatory cytokines, such as interleukin (IL)-1β and tumor necrosis factor (TNF)-α detected through quantitative PCR. Western blot analyses confirmed significant upregulation of Rab8a, VAMP2, and Syntaxin16 in these cells. Furthermore, the application of botulinum neurotoxin type A (BONT/A) reduced the levels of vesicle transport-associated proteins, inhibiting vesicular transport in activated astrocytes. These findings suggest that the Rab8a/SNARE pathway in astrocytes enhances vesicle transport and anchoring, increasing the secretion of bioactive molecules that may play a crucial role in the pathophysiology of NPP. Inhibiting this pathway with BONT/A offers a novel therapeutic target for managing NPP, highlighting its potential utility in clinical interventions.

## Introduction

Neuropathic pain (NPP) represents a global therapeutic challenge characterized by complex pathophysiological mechanisms and a lack of effective clinical analgesics [1–5]. The functional specificity of cortical networks and their projection targets in the pain process occurs at least on four interconnected levels: dynamic activity states within the cortical network; functionally distinct subdomains; specific circuit connections that distinguish pain from other functions; and co-active cell assemblies [6]. Among these, intercellular communication and molecular signaling pathways within specific circuit connections play a pivotal role in the sensitization and regulation of nociceptive pathways in the sensory nervous system and the pathological process of NPP [7–10].

Astrocytes, distinguishable by their expression of glial fibrillary acidic protein (GFAP) across all major branches and processes, dynamically modulate in response to injury through gap junction protein complexes that physically couple adjacent cells, allowing free exchange of ions and cytoplasmic components [11]. Inhibition of astrocyte activation can significantly alleviate pain caused by peripheral nerve damage in the early stages of NPP [12–14]. Astrocytes mediate intercellular communication within the nervous system through the production and secretion of neuroactive substances [15–17]. Injury signals drive phenotypic transformation of astrocytes and the release of inflammatory mediators, playing roles in central and peripheral sensitization and participating in the progression of NPP. An important characteristic of their activation is the increased release of bioactive molecules such as inflammatory factors, ATP, and glutamate [18–24].

Furthermore, astrocytes contain vesicles that store and release bioactive molecules in an activity-dependent manner, a principal mechanism in the pathophysiology of neurodegenerative diseases [25–31]. However, the specific mechanisms by which astrocytes in NPP increase the secretion of bioactive molecules remain unclear [32], complicating the identification of targets for intervention.

Rab proteins, acting as molecular switches in vesicle transport, interact with upstream regulators and downstream effectors, playing a critical role in vesicle movement, docking, and fusion [33, 34]. In their active GTP-bound form, Rab proteins activate downstream effector proteins, recruit cytoplasmic adhesion factors, and regulate vesicle dynamics [35–42]. The fusion of vesicles with the cell membrane also relies on a set of transmembrane proteins known as the soluble N-ethylmaleimide-Sensitive factor attachment protein receptor (SNARE) complex, which provides the molecular basis for directed vesicle transport, targeting, docking, and membrane fusion [43–45]. Currently, the role of Rab8a in vesicle release processes in spinal astrocytes has not been reported. Thus, this study aims to examine the modification of Rab8a in activated astrocytes using a rat model with sciatic nerve ligation and lipopolysaccharide (LPS)-treated primary astrocytes to investigate its role in SNARE complex formation and vesicle transport and to explore the impact of the Rab8a/SNARE signaling pathway on NPP and its mechanisms. By revealing the role of this signaling pathway in regulating astrocyte vesicle transport and secretion functions, we aim to provide a new perspective on the molecular mechanisms of NPP and lay the groundwork for developing targeted therapeutic strategies, which hold significant scientific and clinical relevance.

## Materials and methods

### Experimental animal models

Male Sprague–Dawley (SD) rats, aged 7–8 weeks (200–230 g), were obtained from the Experimental Animal Research Institute of the Army Medical University. These rats were housed in a controlled environment at 25 ^∘^C with a 12-h light/dark cycle, with free access to food and water. The animal experimental processes were approved by the Ethnic Committee of The First Affiliated Hospital of Chongqing Medical University (AMUWEC20210719) and conducted in strict accordance with the standard of the Guide for the Care and Use of Laboratory Animals published by the Ministry of Science and Technology of the People’s Republic of China in 2006.

### Induction of NPP through chronic constriction injury (CCI)

Ten SD rats (aged 7–8 weeks, weighing 200–230 g) were utilized. The sample size calculation was based on setting the range of acceptable degrees of freedom (DF) for analysis of variance (ANOVA) between 10 and 20. Let *N* represents the total number of subjects, *k* represents the number of groups, and *n* represents the number of subjects per group, calculated as *n* ═ DF/*k* + 1. Hence, the minimum total sample size N(min) was determined to be 6, and the maximum total sample size *N*(max) was 11 [46]. The ten rats were randomly divided into two groups: a normal group (control group, *n* ═ 5) and a chronic constriction injury (CCI) group (ligation group, *n* ═ 5). Each group underwent specific procedures: the normal group received a sham operation without ligation; the CCI group was subjected to a procedure established in previous studies [47]. Briefly, a blunt dissection was performed in the biceps femoris, exposing the common sciatic nerve at the mid-thigh level. Approximately 1 cm of the nerve was freed from the surrounding connective tissue near its trifurcation, and three loops of 4.0 non-absorbable surgical suture (Shanghai Fosun) were loosely tied around it at 1 mm intervals. Under 30× magnification, these ties did not significantly compress the nerve’s diameter but did induce slight and transient twitches in the muscles innervated by the sciatic nerve. The test animals were subsequently maintained for 14 days.

### Astrocyte culture

Primary astrocytes were prepared from one-day-old SD rats, following the procedure described by Schildge et al. [48]. These cells were isolated from the cerebral cortex and subsequently cultured in 25 cm^2^ flasks precoated with 50 µg/mL poly-D-lysine. The culture medium used was DMEM (Gibco, New York, USA) supplemented with 10% heat-inactivated fetal bovine serum and 1% penicillin–streptomycin (Beyotime, Shanghai, China). The cultures were maintained under conditions of 5% CO_2_ at 37 ^∘^C. The medium was replaced the day following the initial culture and thereafter every two days. On the seventh day, the cultures were placed on a rotating shaker at 37 ^∘^C for 6 h (240 rpm) to detach microglial and oligodendrocyte precursor cells. Following this, the medium was discarded, and the astrocytes were cultured at a final density of 1.2 × 10^6^ cells per well in 6-well plates and 4 × 10^4^ cells per well in 96-well plates for subsequent cell counting kit-8 (CCK8) assay.

In the drug treatment groups, astrocytes were co-incubated with LPS (100 ng/mL) and botulinum toxin A (BONT/A) (0.1 U/mL) for 1 h [49–52]. In this study, LPS was used as a cell activator and BONT/A as a vesicular secretion inhibitor. Immunofluorescence (IF) staining with GFAP (an astrocyte marker, BM-0055, Bioss, Wuhan, China) was performed to identify the astrocytes. A high-purity population of astrocytes (over 95% GFAP-positive) was obtained [53]. To ensure cell culture quality, high-quality fetal bovine serum and culture medium, along with sterile plastic products designed specifically for tissue culture, were used. To prevent microbial contamination, 100 U/mL penicillin–streptomycin (Bi Yun Tian, C0222) was employed to protect against cellular contamination. Mycoplasma testing was performed prior to experiments to exclude mycoplasma infections.

### Cell viability assay

Cell viability was assessed using CCK-8 (Bioss, Beijing, China). Astrocytes were cultured in 96-well plates for 24 h. Following treatment with LPS (100 ng/mL) for 24 h, CCK-8 solution was added to each well and incubated at 37 ^∘^C for 2 h. Absorbance was measured at 450 nm using a microplate reader.

### Immunohistochemistry (IHC)

Spinal cord tissues from CCI rats were collected on day 14 post-sciatic nerve ligation. Rats were deeply anesthetized with isoflurane (2%–2.5%, airflow 500–700 mL/min) and then perfused intracardially with 4% paraformaldehyde (Sigma) pre-cooled to 4 ^∘^C. The spinal cord was quickly removed and immersed in 4% paraformaldehyde at 4 ^∘^C overnight. After fixation, the spinal cord was dehydrated, and the lumbar enlargement region was sectioned into 16-µm thick slices. Endogenous peroxidase activity was blocked using 3% H_2_O_2_ for 20 min. Sections were incubated with 10% normal goat serum and anti-Rab8a antibody (1:150; LifeSpan Biosciences) at 37 ^∘^C for 1 h, followed by overnight incubation at 4 ^∘^C. After PBS rinsing, sections were incubated at 37 ^∘^C for 1 h and visualized using an enhanced nickel-DAB staining reagent for 5 min. IHC images were captured using a microscope (Leica). Five random spinal cord sections were selected by two experienced pathologists in a blinded manner, and the average optical density of all positively stained astrocytes in the selected fields was measured and analyzed using Image-Pro Plus 6.0.

### Immunofluorescence (IF) staining

For double-labeling IF experiments on spinal cord sections, prepared slices were treated with 3% H_2_O_2_ for 20 min to suppress endogenous peroxidase activity. The sections were then incubated at 37 ^∘^C for 1 h, followed by overnight treatment at 4 ^∘^C with 10% normal goat serum. Subsequently, the slices were incubated with anti-GFAP monoclonal antibody (1:250, Bioss) at 37 ^∘^C for 1 h, followed by co-incubation with anti-Rab8a polyclonal antibody (1:150, LifeSpan Biosciences) at 37 ^∘^C for 1 h, and then overnight at 4 ^∘^C. FITC-conjugated goat anti-mouse antibody (1:500, Abcam, UK) and Cy3-conjugated goat anti-rabbit antibody (1:600, Jackson ImmunoResearch, USA) were added and incubated at 37 ^∘^C for 1 h. Finally, nuclei were stained with 4′,6-diamidino-2-phenylindole (DAPI) (Sigma, USA) and analyzed by two experienced pathologists in a blinded manner using a laser scanning confocal microscope (Olympus, Japan).

For IF staining experiments detecting GFAP in astrocytes, astrocytes grown on microscope slides were fixed in 4% paraformaldehyde at 37 ^∘^C for 30 min, followed by incubation in 5% BSA at 37 ^∘^C for 1 h and then overnight incubated with anti-GFAP monoclonal antibody (1:250, Bioss) at 4 ^∘^C. The cells were then incubated at 37 ^∘^C for 1 h with FITC-conjugated goat anti-mouse antibody (1:500, Abcam, UK). Nuclei were visualized with DAPI staining (Bioss, Beijing, China), and images were captured using a microscope (Leica).

In the double-labeling, IF experiments, astrocytes grown on microscope slides were fixed with 4% paraformaldehyde at 37 ^∘^C for 30 min, then incubated in 5% BSA at 37 ^∘^C for 1 h, followed by overnight incubation with anti-Rab8a polyclonal antibody (1:250, LifeSpan Biosciences) at 4 ^∘^C. FITC-conjugated goat anti-mouse antibody (1:500, Abcam, UK) and Cy3-conjugated goat anti-rabbit antibody (1:600, Jackson ImmunoResearch, USA) were added and incubated at 37 ^∘^C for 1 h. Nuclei were stained with DAPI (Cat# D9542-5MG, Sigma, USA) and analyzed using a laser scanning confocal microscope (Leica).

### Western blot (WB) assay

Cell lysates were collected from primary astrocyte cultures in RIPA buffer containing a protease inhibitor cocktail for Western blot analysis 1-h post-LPS stimulation. The reaction mixtures were centrifuged at 12,000 × *g* for 15 min at 4 ^∘^C. Samples containing 2 µg of protein were heated at 100 ^∘^C for 5 min in a loading buffer (5× Loading Buffer, Beyotime, Shanghai, China). Separation was conducted using polyacrylamide gels (10%–12.5%, Epizyme, Beijing, China). Following membrane transfer, the membranes were incubated overnight at 4 ^∘^C with anti-GFAP monoclonal antibody (1:1000, Bioss), anti-Rab8a polyclonal antibody (1:1000, LifeSpan), anti-VAMP2 polyclonal antibody (1:1000, Cell Signaling), anti-Syntaxin16 polyclonal antibody (1:1000, Cell Signaling), and anti-β-actin (1:1000, Proteintech). The membranes were then incubated for 1 h with horseradish peroxidase-conjugated secondary antibodies and visualized using ECL solution (Biosharp, Shanghai, China). Immunocomplexes were detected using the Bio-Rad system, and relative immunoreactivity levels were quantified using Image Lab software.

### Quantitative real-time polymerase chain reaction (qPCR)

Total RNA from astrocytes was isolated using the RNAeasy™ animal RNA isolation kit with the spin column, following the manufacturer’s instructions (Beyotime, Shanghai, China). RNA sample transcription was repeated using the PrimeScript™ RT reagent kit with gDNA Eraser (Takara, Japan), according to the manufacturer’s instructions. Real-time qPCR was conducted using SYBR Premix Ex Taq II (Takara). The thermal cycling program included a 10-min pre-incubation at 95 ^∘^C, followed by 45 cycles of 10 s at 95 ^∘^C, 30 s at 60 ^∘^C, and 60 s at 65 ^∘^C. The specificity of the PCR products was verified through melt curve analysis.

### Electron microscopy (EM)

Astrocytes were co-incubated with LPS (100 ng/mL) or LPS (100 ng/mL) and BONT/A (0.1 U/mL) for 24 h. Cells were then detached using a 0.025% trypsin-EDTA solution and fixed with 2.5% glutaraldehyde at 4 ^∘^C for 12 h. The prepared cells were further fixed with 1% osmium tetroxide at 4 ^∘^C for 1 h. After gradient dehydration, the cells were embedded in resin. Embedded cell sections were then observed under a transmission EM.

### Ethical statement

The animal experimental processes were approved by the Ethics Committee of Third Military Medical university (AMUWEC20210719) and conducted in strict accordance with the standard of the Guide for the Care and Use of Laboratory Animals published by the Ministry of Science and Technology of the People’s Republic of China in 2006.

### Statistical analysis

All statistical analyses were conducted using version 4.2.1 of R (R Foundation for Statistical Computing). Quantitative data in this study were analyzed using GraphPad Prism version 9.5.0. Data were presented as mean ± standard deviation. Initially, tests for normality and homogeneity of variance were performed. If the data were normally distributed and the variances were homogeneous, unpaired *t*-tests were used to compare differences between the two groups. One-way ANOVA was employed to compare differences among multiple groups, followed by Tukey’s post-hoc test for pairwise comparisons. A *P* < 0.05 was considered statistically significant, while a *P* < 0.01 was considered highly significant.

## Results

### Activation of astrocytes and increased Rab8a expression in the spinal dorsal horn of CCI rats

**Figure 1.**
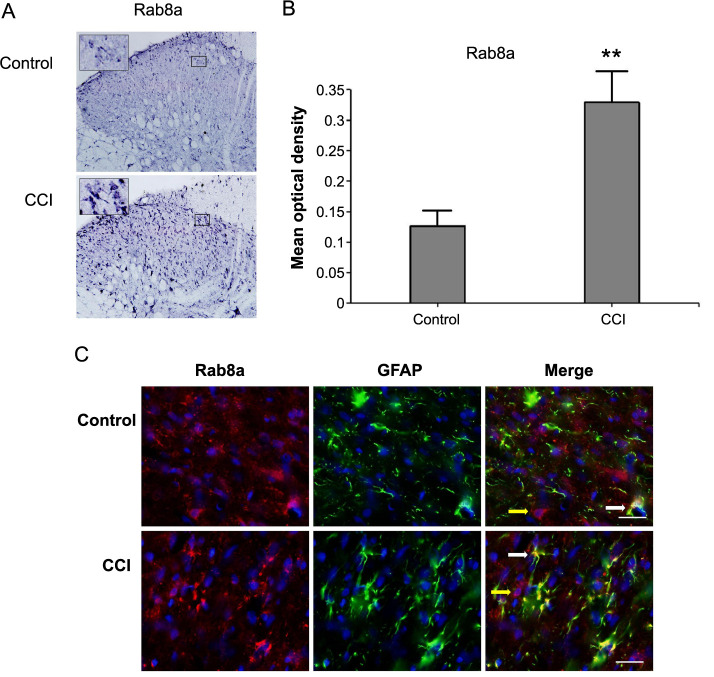
**Activation of astrocytes and Rab8a expression in the spinal dorsal horn of CCI rats.** (A) IHC staining of Rab8a in the spinal dorsal horn of CCI rats; (B) Quantitative analysis of Rab8a protein in the spinal dorsal horn of CCI rats; (C) IF staining in the spinal dorsal horn of CCI rats, showing GFAP-positive cells (green fluorescence) and the distribution of Rab8a (red fluorescence). **P < 0.01. CCI: Chronic constriction injury; IHC: Immunohistochemistry; GFAP: Glial fibrillary acidic protein.

In our study of the sciatic nerve ligation model in rats, we conducted IHC and IF staining to investigate the potential mechanisms related to astrocytes in NPP. IHC analysis revealed a significant increase in Rab8a expression in the spinal dorsal horn of rats subjected to sciatic nerve ligation compared to controls (Figure 1A and 1B, *P* < 0.01).

IF staining further explored the distribution of Rab8a in the spinal dorsal horn. GFAP (green fluorescence), a marker of astrocytes, showed a notable increase in the NPP model, indicating the activation of astrocytes. Rab8a (red fluorescence) staining was observed in various cell types within the spinal dorsal horn, but a significant increase in Rab8a expression was evident in activated astrocytes (Figure 1C). These findings highlight the association between Rab8a expression and astrocyte activation, suggesting its potential importance in the pathophysiology of NPP.

### Upregulation of cytokine expression in LPS-induced activated astrocytes

**Figure 2.**
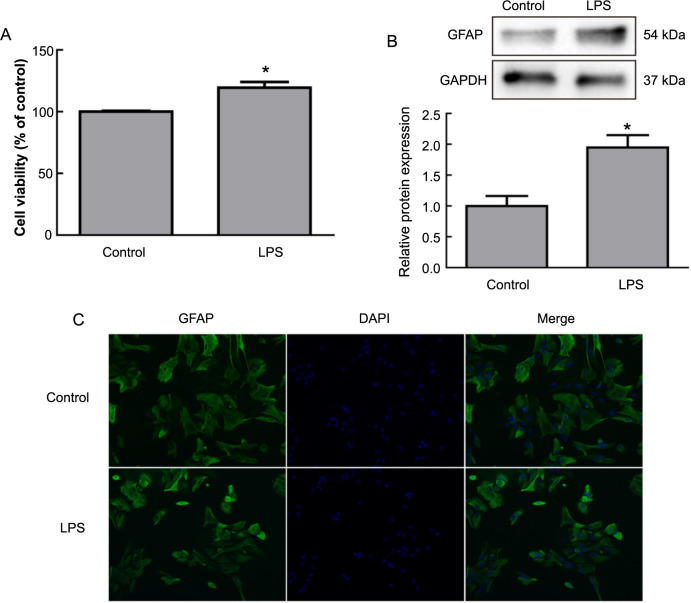
**The effects of LPS on astrocytes.** (A) The survival rate of astrocytes post-LPS treatment; (B) GFAP protein expression in astrocytes post-LPS treatment detected by Western blot; (C) IF staining of astrocytes post-LPS treatment, showing GFAP-positive cells (green fluorescence) and morphological changes. *P < 0.05. GFAP: Glial fibrillary acidic protein; LPS: Lipopolysaccharide; IF: Immunofluorescence.

LPS is commonly utilized to simulate inflammatory responses, prompting a series of experiments to investigate its effects on astrocytes. Initially, we assessed the viability of astrocytes to gauge the activating effect of LPS. The results demonstrated a significant increase in the survival rate of astrocytes cultured with LPS compared to the control group (Figure 2A, *P* < 0.05).

IF and Western blot analyses revealed a significant increase in the expression of the GFAP protein in cells treated with LPS (Figure 2B, *P* < 0.05). Moreover, compared to the control group, cells in the LPS group exhibited increased cell volume and shorter, thicker processes (Figure 2C). This phenomenon likely reflects the morphological changes of astrocytes under LPS treatment, further supporting their activated state.

**Figure 3.**
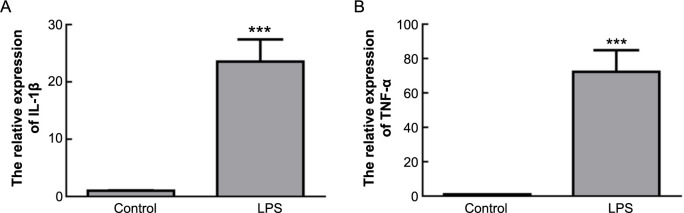
**Expression of cytokines in activated astrocytes.** (A) qPCR detection of TNF-α mRNA levels in astrocytes post-LPS treatment; (B) qPCR detection of IL-1β mRNA levels in astrocytes post-LPS treatment. ***P < 0.001. LPS: Lipopolysaccharide; qPCR: Quantitative real-time polymerase chain reaction.

Further analysis through qPCR was conducted to measure the mRNA levels of pro-inflammatory cytokines, including TNF-α and IL-1β, in activated astrocytes. The results showed significant upregulation of TNF-α and IL-1β mRNA levels in activated astrocytes (Figure 3A and 3B, *P* < 0.001).

In summary, our findings reveal the activating effects of LPS on astrocytes, including increased cell viability, elevated expression of GFAP protein, morphological changes, and the upregulation of pro-inflammatory cytokines TNF-α and IL-1β mRNA levels in activated astrocytes.

### Increased vesicular transport in LPS-induced activated astrocytes

**Figure 4.**
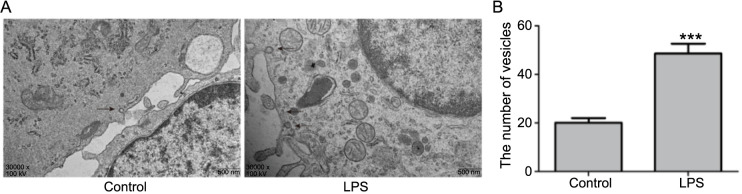
**Changes in vesicular transport in activated astrocytes.** (A) EM observation of vesicular transport in astrocytes post-LPS treatment; (B) Statistical graph of vesicular transport quantity in astrocytes post-LPS treatment. ***P < 0.001. EM: Electron microscopy; LPS: Lipopolysaccharide.

To investigate changes in vesicular transport within activated astrocytes, EM was employed to examine vesicular transport. In the LPS-treated group, the Golgi apparatus was increased and enlarged, with more Golgi vesicles around the trans-Golgi network (TGN). Mitochondrial numbers were increased, showing swollen, spherical forms with reduced cristae. The quantity of vesicles in activated astrocytes was higher in the LPS group, with vesicles accumulating near the cell membrane (Figure 4A and 4B).

**Figure 5.**
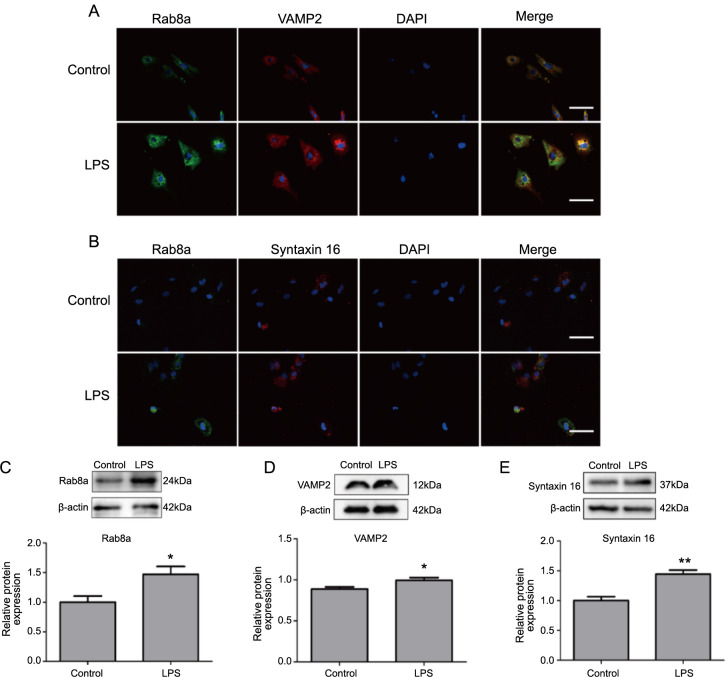
**Levels of vesicular transport-related proteins in activated astrocytes.** (A and B) IF staining of VAMP2 and Syntaxin16 in astrocytes post-LPS treatment; (C–E) Western blot detection of Rab8a and SNARE proteins (VAMP2 and Syntaxin16) levels in astrocytes post-LPS treatment. *P < 0.05, **P < 0.01. IF: Immunofluorescence; VAMP2: Vesicle-associated membrane protein; SNARE: Soluble N-ethylmaleimide-sensitive factor attachment protein receptor; LPS: Lipopolysaccharide.

Given the molecular basis of vesicle and plasma membrane fusion established by the SNARE complex [44], the co-expression of Rab8a and VAMP2 in activated astrocytes was further investigated through IF experiments. It was observed that the positive IF staining of Rab8a and VAMP2 was more aggregated in activated astrocytes. Additionally, astrocytes activated by LPS also exhibited co-localization of Rab8a and VAMP2 expression, with these proteins displaying a relatively uniform distribution across various cell types (Figure 5A).

Furthermore, co-localization of Rab8a and Syntaxin16 expression was also observed in astrocytes activated by LPS. However, the distribution of the positive IF staining for Rab8a and Syntaxin16 was not entirely consistent across different activated astrocytes (Figure 5B).

Western blot analysis further examined the expression levels of vesicular transport-related proteins (Rab8a, VAMP2, and Syntaxin16). The results indicated that, following LPS treatment, the levels of Rab8a, VAMP2, and Syntaxin16 proteins were significantly higher in astrocytes compared to the control group (Figure 5C–5E, *P* < 0.05).

These findings reveal increased vesicular transport in LPS-induced activated astrocytes, accompanied by upregulation of protein levels of Rab8a and SNARE.

**Figure 6.**
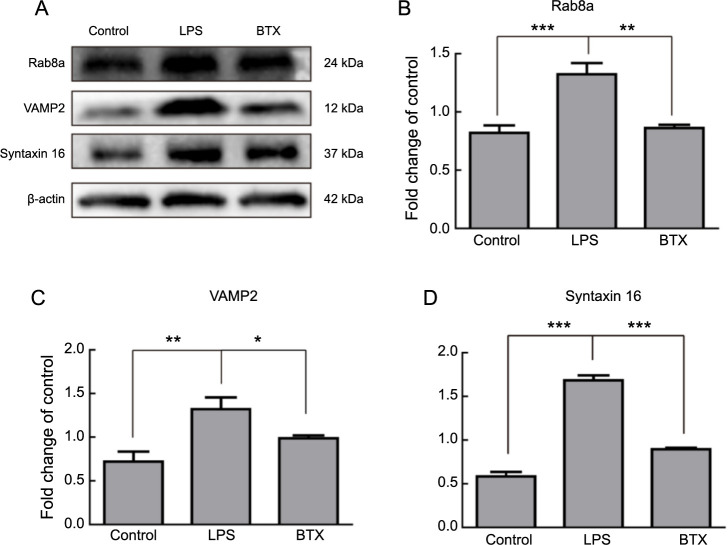
**Effects of BONT/A on vesicular transport-related proteins in activated astrocytes.** (A) Western blot detection of Rab8a, VAMP2, and Syntaxin16 protein expression in activated astrocytes post-BONT/A treatment; (B–D) Quantitative analysis of Rab8a, VAMP2, and Syntaxin16 proteins in activated astrocytes post-BONT/A treatment. *P < 0.05, **P < 0.01, ***P < 0.001. BONT/A: Botulinum neurotoxin type A; LPS: Lipopolysaccharide; VAMP2: Vesicle-associated membrane protein; BTX: Botulinum toxin.

**Figure 7.**
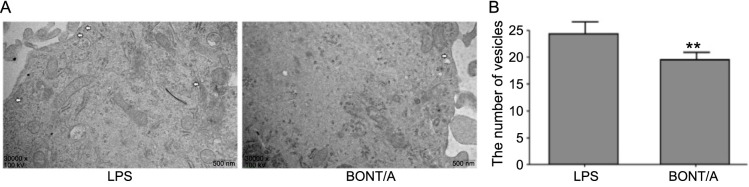
**Effects of BONT/A on vesicular transport in activated astrocytes.** (A) EM observation of vesicular transport in activated astrocytes post-BONT/A treatment; (B) Statistical analysis of vesicular transport quantity in activated astrocytes post-BONT/A treatment. **P < 0.01. BONT/A: Botulinum neurotoxin type A; EM: Electron microscopy; LPS: Lipopolysaccharide.

### BONT/A inhibits vesicular transport in LPS-induced activated astrocytes

To assess the impact of the vesicular secretion inhibitor BONT/A on vesicular transport, we conducted Western blot experiments to detect changes in proteins related to vesicular transport (Rab8a, VAMP2, and Syntaxin16) in astrocytes activated by LPS treatment following BONT/A administration. The results indicated a significant reduction in the expression levels of Rab8a, VAMP2, and Syntaxin16 proteins in astrocytes treated with BONT/A (BTX group) compared to those treated with LPS alone (LPS group) (Figure 6A–6D).

To gain a comprehensive understanding of BONT/A’s effect, EM was used to evaluate changes in vesicular transport. The findings demonstrated that vesicular transport within astrocytes activated by LPS was significantly inhibited following BONT/A treatment, evidenced by a decrease in the number of intracellular vesicles and a marked reduction in vesicle accumulation near the cell membrane (Figure 7A and 7B).

These results reveal the inhibitory effect of BONT/A on vesicular transport in astrocytes activated by LPS induction.

## Discussion

Our research demonstrates that injury signals drive the transformation and activation of astrocytes, leading to increased release of pain-associated bioactive molecules, such as inflammatory factors, ATP, and glutamate. These molecules play roles in central and peripheral sensitization and contribute to the progression of NPP [18, 19]. Astrocyte activation is a heterogeneous process involving multiple molecular, cellular, and functional changes, including alterations in vesicular secretion [25, 54–56]. However, the specific mechanisms underlying vesicle and inflammatory mediator release remain unclear [32]. Rab8a protein and the SNARE complex are involved in vesicle-directed transport, targeting docking, and fusion with the cell membrane [43–45], but their mechanisms in NPP have yet to be confirmed.

**Figure 8.**
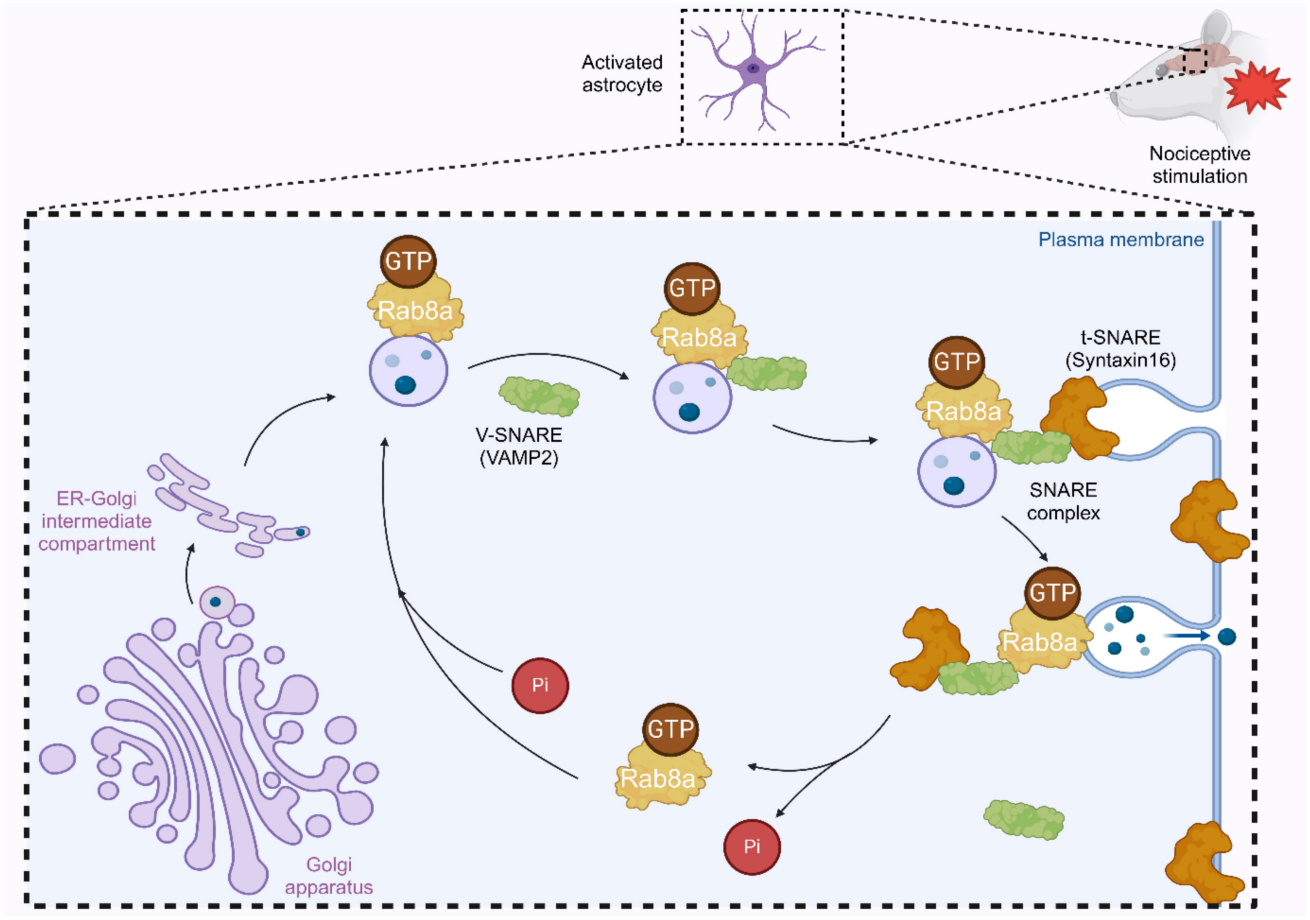
**Activated Rab8a/SNARE complex drives the molecular mechanism of NPP by promoting vesicle anchoring and transportation in spinal astrocytes.** NPP: Neuropathic pain; GTP: Guanosine triphosphate; VAMP2: Vesicle-associated membrane protein; V-SNARE: Vesicle soluble N-ethylmaleimide-sensitive factor attachment protein receptor; t-SNARE: Target soluble N-ethylmaleimide-sensitive factor attachment protein receptor.

In our study using a CCI rat model, Rab8a was highly expressed in astrocytes within the spinal dorsal horn following neural injury, suggesting increased vesicle docking and transport activity, a possible manifestation of astrocyte activation. EM revealed a significant increase in internal vesicle number and transport activity toward the plasma membrane, resulting in heightened exocytic activity. Quantitative PCR, IF, and Western blot results showed significant increases in the expression of cytokines, such as TNF-α and IL-1β, as well as Rab8a, VAMP2, and Syntaxin16 in activated astrocytes. Treatment with BONT/A significantly reduced the levels of Rab8a, VAMP2, and Syntaxin16 proteins in astrocytes. Collectively, these findings suggest that the activation of the Rab8a/SNARE complex pathway is crucial for vesicular transport and bioactive molecule release in astrocytes and represents an important component in the pathogenesis of NPP.

Rab8a, a small GTPase, is essential for vesicle transport in various cell types and is involved in cilia formation [57, 58]. Rab8a can interact with effectors or directly with SNARE to recognize t-SNARE on target membranes, promoting v-SNARE and t-SNARE pairing, thus guiding vesicle-directed transport and targeted docking [59–64]. The control of vesicle transport by Rab8a may facilitate the formation of different membrane protrusions, while VAMP2 and Syntaxin16, components of the SNARE complex, are critical proteins in vesicle docking. Our results, combined with previous studies [65, 66], suggest that Rab8a-mediated enhanced transport of vesicles from the TGN to the plasma membrane may underpin the molecular basis for astrocyte release of bioactive molecules involved in the onset and maintenance of NPP. Enhanced vesicular transport in LPS-activated astrocyte models likely represents a crucial mechanism for the secretion of bioactive molecules by activated astrocytes, with activated pathways for cytokine synthesis and secretion contributing to disease progression.

Furthermore, the application of BONT/A suggests that targeting components of the SNARE complex can effectively reduce vesicular transport in astrocytes. Preclinical and clinical studies have reported the efficacy of BONT/A in treating central NPP. BONT/A inhibits the secretion of substance P and calcitonin gene-related peptide (CGRP) in DRG, suppresses the expression of TRPV1 and P2X3, and exerts central effects through retrograde axonal transport [67–70]. BONT/A not only cleaves SNAP-25 at presynaptic terminals but also cleaves SNARE proteins retrogradely in growth cones and the central brain, inhibiting the exocytosis of vesicles containing norepinephrine, glutamate, substance P, and CGRP, as well as the expression of vanilloid receptors.

Although this study provides important insights into the role of the Rab8a/SNARE complex in NPP, it has limitations. Firstly, the study is primarily based on animal models and cell experiments, and its results need further validation in humans. Secondly, although BONT/A can inhibit vesicular transport in astrocytes, its specific mechanisms of action and long-term effects require further investigation. Additionally, this study did not fully resolve all potential molecular mechanisms of the Rab8a/SNARE complex pathway in the pathogenesis of NPP, necessitating further research to elucidate these mechanisms.

## Conclusion

In summary, our study reveals that the activation of the Rab8a/SNARE complex pathway and subsequent enhanced vesicle transport activity in the spinal dorsal horn following neural injury are likely critical components in the cytokine cascade reaction mechanisms of NPP (Figure 8). By elucidating the role of the Rab8a/SNARE complex in the development of NPP, this study provides important insights for understanding the molecular basis of NPP and developing new therapeutic strategies. Given the persistent activation of astrocytes under chronic pain conditions and their recognized role in NPP, directing therapeutic interventions toward reactive astrocytes holds significant potential. Our research demonstrates the critical role of these proteins in astrocytes and emphasizes the importance of vesicle transport in regulating NPP, offering new potential targets for NPP treatment. Targeting the Rab8a/SNARE complex pathway could be an effective strategy for alleviating or treating NPP. Based on our current understanding of astrocyte-mediated NPP, considering targeting related signaling pathways, hemichannels, or purinergic receptors to inhibit the release of neuroglial mediators, such as by inhibiting the expression or function of Rab8a to reduce the release of inflammatory mediators, could provide valuable directions for developing novel NPP therapeutic drugs. Additionally, targeting downstream mediators released by astrocytes, such as chemokines and cytokine signaling, is a viable treatment strategy. Given that astrocyte dysregulation is a common feature of nearly all chronic pain pathologies, and the activation of astrocytes remains strong throughout persistent pain conditions, whether targeting the activation of astrocytes or preventing their transition to a pro-inflammatory state without affecting their normal homeostatic functions remains a significant challenge.

Future research should focus on several key areas. Firstly, the findings of this study need to be validated in a broader range of biological models and explored through clinical studies to assess their potential application in human NPP treatment. Secondly, specific intervention methods targeting the Rab8a/SNARE complex pathway, including small molecule inhibitors, and RNA interference techniques, should be explored to develop new treatment strategies. Additionally, investigating the role of the Rab8a/SNARE complex in other cell types beyond astrocytes, such as neurons and microglia, may reveal more complex pathological mechanisms of NPP.

## Data Availability

Data will be made available on request.
