# Erratum: Rab8a/SNARE complex activation promotes vesicle anchoring and transport in spinal astrocytes to drive neuropathic pain

**DOI:** 10.17305/bb.2025.12773

**Published:** 2025-04-07



In the article “***Rab8a/SNARE complex activation promotes vesicle anchoring and transport in spinal astrocytes to drive neuropathic pain***” (Xiao et al., DOI: https://doi.org/10.17305/bb.2024.10441), published on 6 September 2024, an incorrect version of Figure 2 was inadvertently published due to an editorial oversight. The article has been corrected online to include the accurate figure. This correction does not affect the study’s results, interpretations, or conclusions. We apologize for the error and thank the authors for bringing it to our attention.

